# The effect of temporal aggregation level in social network monitoring

**DOI:** 10.1371/journal.pone.0209075

**Published:** 2018-12-19

**Authors:** Meng J. Zhao, Anne R. Driscoll, Srijan Sengupta, Nathaniel T. Stevens, Ronal D. Fricker, William H. Woodall

**Affiliations:** 1 Department of Statistics, Virginia Polytechnic Institute and State University, Blacksburg, VA, United States of America; 2 Department of Mathematics and Statistics, University of San Francisco, San Francisco, California, United States of America; Universidad de las Palmas de Gran Canaria, SPAIN

## Abstract

Social networks have become ubiquitous in modern society, which makes social network monitoring a research area of significant practical importance. Social network data consist of social interactions between pairs of individuals that are temporally aggregated over a certain interval of time, and the level of such temporal aggregation can have substantial impact on social network monitoring. There have been several studies on the effect of temporal aggregation in the process monitoring literature, but no studies on the effect of temporal aggregation in social network monitoring. We use the degree corrected stochastic block model (DCSBM) to simulate social networks and network anomalies and analyze these networks in the context of both count and binary network data. In conjunction with this model, we use the Priebe scan method as the monitoring method. We demonstrate that temporal aggregation at high levels leads to a considerable decrease in the ability to detect an anomaly within a specified time period. Moreover, converting social network communication data from counts to binary indicators can result in a significant loss of information, hindering detection performance. Aggregation at an appropriate level with count data, however, can amplify the anomalous signal generated by network anomalies and improve detection performance. Our results provide both insights on the practical effects of temporal aggregation and a framework for the study of other combinations of network models, surveillance methods, and types of anomalies.

## Introduction

The availability of network data has increased dramatically in the last decade or so due to developments in communication technology. The origins of these data vary greatly depending on sources such as cell phone networks, social media, and other internet-based communications. Researchers face difficult challenges due to the velocity and the volume at which these data are generated. Fittingly, statistical analysis of networks has recently received increased emphasis in the statistics literature, leading to the development of a rich toolbox of network models and inference methods. Broad reviews of statistical network analysis can be found in Kolaczyk [1], Goldenberg et al. [2], and Salter-Townshend et al. [3].

The fundamental goal of social network monitoring or surveillance is to detect sudden and significant changes in the communication patterns of a group of individuals. This is a topic of significant practical importance, since such significant changes can have serious implications like societal change, predatory activity, disruptive or fraudulent behavior, etc. However, as pointed out by Woodall et al. [4], how to best monitor social networks has received proportionately less attention from the statistics community in spite of its exceptional relevance in today’s interconnected world. In this article we aim to partially address this gap.

The goal of social network monitoring is to detect anomalous behavior in a network in a timely fashion. In the social network monitoring methods reviewed by Savage et al. [5], Bindu and Thilagam [6] and Woodall et al. [4], the data either consist of counts of communications between pairs of individuals or indicator variables indicating the presence or absence of a specified threshold level of communication. Data are typically aggregated over a given time interval such as day or week. The length of the aggregation interval affects the amount of information that is contained within the reported data. Therefore, the level of temporal aggregation impacts the performance of any monitoring technique. Zwetsloot and Woodall [7] provide a comprehensive review of the literature on the effect of temporal aggregation on process monitoring performance, though they do not consider social network monitoring applications. In our paper, we study the effect of temporal aggregation and the effect of conversion of count data to binary data on social network surveillance using a particular model and surveillance method.

Zwetsloot and Woodall [7] stated that aggregating data over time results in a loss of information and can slow detection of process changes. Our investigation shows that this information loss can be substantial if the social network data are converted from communication counts to binary indicators. The loss is, however, is not as severe when communication data are recorded as count data. We provide insights and a framework for studies with more sophisticated models. Additionally, we show that aggregation in certain situations can amplify the anomalous events in the social network, thereby improving the detection sensitivity of the method used. Nevertheless, our study reveals that too much aggregation can degrade the detection performance by delaying a signal of the presence of an anomaly, a finding which is consistent with Zwetsloot and Woodall [7].

From a practical perspective, communication rates in a social network often vary regularly over the course of a day or some other time period. Aggregating by hour when there is regular seasonal variation within the day would require additional modeling. Aggregating the data by day would greatly simplify the modeling and analysis by making the successive networks more time-stationary. We assume in our paper that the most basic level of aggregation has removed any seasonal variation.

Beginning from the pioneering work by Erdős and Rényi [8], there has been a progression of increasingly more realistic random graph models in the statistics literature. Lorrain and White [9] proposed the idea of blockmodels for networks with community structure, which was fully developed by Holland et al. [10] and Fienberg et al. [11] leading to the formulation of the stochastic blockmodel (SBM). In recent years, a comprehensive set of statistical estimation and inference methods have been formulated using the SBM, with notable contributions from many authors [12–16].

The SBM is based on the principle of stochastic equivalence, where any two nodes in the same community have the same expected number of connections. This leads to unrealistic constraints since empirical networks exhibit widely varying degrees even for nodes in the same community. To address this issue, Karrer and Newman [17] developed the degree corrected stochastic block model (DCSBM) that allows nodes in the same community to have different expected degrees. The DCSBM has now been established as the current state-of-the-art blockmodel for networks, with notable contributions from Zhao et al. [18], Qin and Rohe [19], Lei and Rinaldo [20], and Jin [21], to name a few. This model has been used by other researchers in network surveillance applications, see Wilson et al. [22] and Yu et al. [23].

The main contribution of our paper is that we carry out a systematic investigation of the effect of aggregation level on social network monitoring. We consider a popular network model, namely the DCSBM model, in conjunction with a popular monitoring method, namely the Priebe scan method of [24] to carry out this investigation. Using this combination, we discovered that the level of temporal aggregation has a significant impact on the effectiveness of anomaly detection. It is important to add the caveat that our specific findings are under this particular combination of network model and monitoring method. However, in general the effect of temporal aggregation level is likely to be significant for social networks generated from any network model, as well as any monitoring method that is used for anomaly detection. The conceptual framework of this paper can be readily extended to study the effect of temporal aggregation under other network models or other monitoring methods.

The rest of the paper is organized as follows: we provide a brief review of some network terminology, followed by a description of the DCSBM model used to simulate the social network data, as well as the scan method used to monitor the networks. Then we introduce the simulation scenarios studied and present the false alarm rates resulting from data aggregated at different levels. Simulation results showing the effect of aggregation level are then presented, as well as the results from studying the conditional signal delays resulting from different levels of temporal aggregation. At the end of the paper, we draw our conclusions and discuss future directions for new work.

## Some network terminology

Here we provide an overview of relevant network terminology and notation to aid readers for whom this is unfamiliar. A social network can be represented by a *graph G* = (*V*,*E*) that consists of a set of *nodes V* and a set of *edges E*. In a social network, a node usually represents an individual or the origin/destination of a communication. When the nodes in a social network contain information that makes them readily identifiable, such as a name or an e-mail address, the social network is said to be *labeled*. If no identifying information is available, it is an *unlabeled* social network. Depending on the type of network data, the edges in the social network specify the level of communication between each pair of nodes over a given period of time. For network count data, the edges represent the number of communication instances between each pairs of nodes. For network binary data, each edge indicates whether or not communication between a pair of nodes exceeds a certain threshold. We denote the number of nodes and edges by |*V*| and |*E*|, respectively. The number of nodes, |*V*|, is referred to as the *order* of the network. The number of edges, |*E*|, is referred to as the *size* of the network.

The communication levels between all pairs of nodes at time *t* for a network *G*_*t*_ = (*V*,*E*_*t*_) are summarized by an adjacency matrix *C*_*t*_ of dimension |*V*|×|*V*|. The matrices *C*_*t*_ are available at the current time (*t* = *T*) and all previous time points, *t* = 1,2,…,T−1, each representing a snapshot of the social network structure at these specific times. The entry of *C*_*t*_ in row *i* and column *j*, denoted by *C*_*t*_(*i*,*j*), is a record of the communication level between nodes *i* and *j* at time point *t*. Note that we do not accommodate self-loops and so the values on the diagonal are set to zero. Networks for which the direction of communications are known are referred to as *directed* networks. When the direction of communication is not specified or unknown, we have *C*_*t*_(*i*,*j*) = *C*_*t*_(*j*,*i*) and *C*_*t*_ is a symmetric matrix. Networks in this case are referred to as *undirected* networks, which are the focus of our investigation. When only the presence or absence of a specified level of communication is measured, each *C*_*t*_(*i*,*j*) value is restricted to 1 (presence) or 0 (absence). When the total number of communications is measured, *C*_*t*_(*i*,*j*) can take non-negative integer values.

The *k*^*th*^ *neighborhood* of node *i* contains all nodes and edges that are within *k* edges of node *i*,*k* = 1,2,3,…. The *size* of the *k*^*th*^ neighborhood of node *i* is the count of all edges between nodes in the *k*^*th*^ neighborhood of node *i*. At time *t*, this quantity is denoted by $O_{t,i}^{k}$. We use the quantity $O_{t,i}^{0}$ to represent the *degree* of node *i*, i.e., the number of communications to or from node *i* at time *t*.

During normal communication, a stream of adjacency matrices can be used to quantify the baseline level of communication among nodes or communities. Any significant deviations in either direction from these baseline levels thereafter is defined as an anomaly. The presence of any anomaly involving node *i* at time *t* is directly reflected by a change in the quantity $O_{t,i}^{k}$. More often than not, unexplained increases in network activities are of interest. These anomalies can represent an uptick in terrorist communications in military or police applications, or opportunities for targeted advertising to a particular social network community for an advertising agency. Although detecting increases in communication among nodes in a sub-network is often of interest, network surveillance techniques may also be used to identify significant decreases in communication if this is of interest.

Priebe’s scan method [24] compares newly observed communications to the established baseline levels in the network. Furthermore, it uses moving windows, in which our understanding of “baseline” behavior is updated as time progresses, to account for the natural dynamic behavior of the network.

## Network model and surveillance method

In this section we describe the network model and surveillance method we use to evaluate the effects of aggregation on social network monitoring.

### Degree corrected stochastic block model

Under the DCSBM model, the node set *V* of a social network is partitioned into *R* disjoint communities where community *r*,*r* = 1,2,…,*R*, contains |*V*_*r*_| nodes and *V* = *V*_1_∪*V*_2_∪⋯∪*V*_*R*_. For the DCSBM model the parameters *θ*_*i*_>0, *i* = 1,…,|*V*|, define the propensity of node *i* to communicate with any other node. The *R*×*R* matrix *P* defines the propensity for communication between the different communities over a given period of time, where element *P*_*r*,*r*′_,{*r* = 1,…,*R* and *r*′ = 1,…,*R*} is the communication propensity between communities *r* and *r*′. Conditional on community membership, the number of communications between nodes *i* and *j* is assumed to be Poisson distributed with mean

$$\lambda_{i,j}=\theta_{i}\theta_{j}P_{r,r^{\prime }},$$ (1)

provided one of the nodes is in community *r* and the other node is in community *r*′. It is important to note that constraints on either the *θ*_*i*_ values or the matrix *P* are required in order to make the model identifiable. Here we use the constraint

$$\sum_{i=1}^{\left|V\right|}\theta_{i}\times I\{node\;i\in community\;r\}=|V_{r}|$$ (2)

*r* = 1,2,…,*R*. Thus the (*i*, *j*) element in the adjacency matrix *C*_*t*_ at time *t* is generated such that

$$C_{t}(i,j)∼Poisson(\lambda_{i,j}).$$ (3)

The adjacency matrix *C*_*t*_ summarizes communications within the social network aggregated over the time period (*t*-1, *t*).

Links in a social network often form as a result of communications taking place between pairs of nodes on a continuous time scale. However, the Priebe method is designed for dynamic networks where network communications are aggregated over sequential time increments. It is interesting to note that if communications occurring in continuous time between nodes *i* and *j* follow a homogeneous Poisson process with an average time between communication of 1/*λ*_*i*,*j*_, then the number of communications during a unit interval of time will follow a Poisson distribution with mean *λ*_*i*,*j*_, which leads to the model in Eq (3). Therefore, even when the underlying communications happen in continuous time, the DCSBM can be interpreted as a discretized version of the resultant network being formed as a result of such communications.

### Priebe’s scan method

The goal of social network monitoring is to detect significant changes in communication patterns in social networks, e.g., whether there has been a significant increase/decrease in the rate of communication among certain groups of individuals. Priebe’s scan method [24] is a popular network monitoring tool for detecting such changes in social networks. This method is based on three sequences of moving window-based scan statistics, the degree, $O_{t,i}^{0}$, and the first two neighborhood sizes, $O_{t,i}^{1}$ and $O_{t,i}^{2},$ across all nodes. The statistics are calculated using a two-step standardization procedure within moving windows of width 20. The standardized statistics of the degree and the first two neighborhood sizes, $O_{t,i}^{k*},$ are calculated in the first standardization procedure as

$$O_{t,i}^{k*}=\frac{O_{t,i}^{k}–avg(O_{t,i}^{k})}{max(sd(O_{t,i}^{k}),1)}$$

where

$$avg(O_{t,i}^{k})=\frac{1}{20}\sum_{j=1}^{20}O_{t-j,i}^{k},and$$

$$sd(O_{t,i}^{k})={\left[\frac{1}{19}\sum_{j=1}^{20}{\left\{O_{t-j,i}^{k}–avg(O_{t,i}^{k})\right\}}^{2}\right]}^{\frac{1}{2}}$$

for *k* = 0,1,2, and 𝑖 = 1, 2, …, |*V*|. Following this, the statistics $M_{t}^{0},\;M_{t}^{1}$, and $M_{t}^{2}$ are calculated as the respective maxima of $O_{t,i}^{0*},\;O_{t,i}^{1*}$, and $O_{t,i}^{2*}$, i.e., $M_{t}^{k}=\max\left\{O_{t,1}^{k*},O_{t,2}^{k*},\ldots ,O_{t,|V|}^{k*}\right\}$. Then $M_{t}^{0*},\;M_{t}^{1*}$, and $M_{t}^{2*}$ are calculated from $M_{t}^{0},\;M_{t}^{1}$, and $M_{t}^{2}$ values using the following equations in a second round of standardization:

$$M_{t}^{k*}=\frac{M_{t}^{k}-avg(M_{t}^{k})}{max(sd(M_{t}^{k}),1)},$$

where

$$avg(M_{t}^{k})=\frac{1}{20}\sum_{j=1}^{20}M_{t-j}^{k},and$$

$$sd(M_{t}^{k})={\left[\frac{1}{19}\sum_{j=1}^{20}{\left\{M_{t-j}^{k}-avg(M_{t}^{k})\right\}}^{2}\right]}^{\frac{1}{2}},$$

for *k* = 0,1,2. The network is monitored over time using $M_{t}^{0*},\;M_{t}^{1*}$, and $M_{t}^{2*}$. The presence of an anomaly is signaled when $max{(M}_{t}^{0*},\;M_{t}^{1*},\;M_{t}^{2*})$ exceeds a pre-specified signaling threshold. The intuition behind this decision rule is that increased communication rates in the network should lead to significant increases in node degrees and neighborhood sizes, resulting in high values of the statistics $M_{t}^{0*},\;M_{t}^{1*}$, and $M_{t}^{2*}$.

Zhao et al. [25] have shown that 4, rather than the value 5 recommended by Priebe et al. [24], is a reasonable signaling threshold. Thus, a signal indicating the possible presence of an anomaly is generated at time *t* if ${max(M}_{t}^{0*},\;M_{t}^{1*},\;M_{t}^{2*})\geq 4$. Although Priebe’s scan method was proposed for binary data, we also use it with count data where we show it can be more effective. Because this method is based on a moving window approach, anomalies become harder to detect if not detected immediately. If any anomaly is not detected within twenty adjacency matrices (i.e., time aggregation periods), the anomalous behavior effectively becomes the new baseline behavior.

## Description of simulation

Woodall et al. [4], Savage et al. [5], Zhao et al. [25] and Fricker [26] all argued that the use of synthetic data is necessary when studying network monitoring methods. We agree with this argument because the temporal information of network anomalies within any existing set of network data can be difficult to determine at best. On the other hand, by using simulations, we have precise control over network size, expected communication rates, the aggregation levels applied to the network data, as well as the severity of the anomaly, which enables us to carry out a systematic investigation. Therefore, following a simulation strategy is better suited than the use of real data for investigating the impact of temporal aggregation on social network monitoring.

Community structure is a prominent feature of the DCSBM, and there are several well-studied community detection methods for estimating this community structure [18, 19, 21, 27]. However, in our investigation, community detection would add noise to the estimated model, which would make it harder to interpret our conclusions regarding the impact of temporal aggregation. Therefore, for simplicity, we assumed in our simulations that the social networks have known community structure. For each node *i* the unscaled value of parameter *θ*_*i*_, denoted as $\theta_{i}^{\prime }$, was generated from a Pareto distribution at the beginning of each simulation run and then held constant during the run, i.e.,

$$\theta_{i}^{\prime }∼iid\;PAR(m=1,s=3)$$

for *i* = 1,…,|*V*|, where *m* denotes the scale parameter and *s* denotes the shape parameter. The Pareto distribution was chosen for its ability to represent degree heterogeneity with skewed degree distributions, as observed in many empirical networks [28, 29]. These values were then scaled to *θ*_*i*_ using the following constraint for nodes within the same community,

$$\theta_{i}=\frac{\theta_{i}^{\prime }}{\sum_{i=1}^{\left|V_{r}\right|}\theta_{i}^{\prime }}\times |V_{r}|.$$

Studies, such as McPherson et al. [30] and Krivitsky et al. [31], have shown that social networks often exhibit homophily, meaning that an individual has a greater tendency to communicate with similar individuals than with dissimilar individuals. We model this in our simulated networks by ensuring that nodes within the same community have a higher propensity to communicate with one another than with nodes in other communities. Thus, the values of the diagonal elements of *P*, *P*_*r*,*r*_ are larger than the values of the off-diagonal elements, *P*_*r*,*r*′_. The ratio between *P*_*r*,*r*_ and *P*_*r*,*r*′_ was kept constant at 2:1 in the simulations, meaning that communications within the same community are twice as likely as communications between different communities. Moreover, low *λ*_*i*,*j*_ values result in networks with low communication levels, or sparse networks. Non-sparse networks with high communication levels are generated by values of *λ*_*i*,*j*_ that are larger.

We performed our simulations using temporal aggregation at five different levels, W = 1, 2, 5, 10, and 20, where we simply summed sequences of adjacency matrices appropriately. For temporal aggregation at level W, the aggregated communications between node *i* and *j* are

$$C_{t}^{\left(W\right)}(i,j)=\sum_{k=t-W+1}^{t}C_{k}(i,j),\;t=W,2W,3W\ldots .$$

In order to establish the moving windows, Priebe’s scan method requires a minimum of 40 adjacency matrices. To ensure that there are enough data for Priebe’s scan method, which requires a minimum of 40 adjacency matrices, a total of 860 *C*_*t*_ adjacency matrices were generated for each round of simulation, where for W = 20, these were then reduced to a sequence of 43 $C_{t}^{\left(20\right)}$ aggregated matrices. Of course, we could have generated the aggregated adjacency matrices directly using the appropriate Poisson distribution, but our approach of summing adjacency matrices removes the possibility that observed performance differences are due to a stochastic variation. In network surveillance, the time period where the network communication baseline is established is referred to as *Phase I*. The *Phase I* in our simulations contain a minimum of 40 adjacency matrices due to the two moving windows required by Priebe’s scan method. *Phase II* refers to the period where real time monitoring occurs. During *Phase II*, a signal is given if communication behavior in the network appears to have deviated significantly from the baseline level. Recall that Priebe’s scan method involves a moving window, which corresponds to a moving baseline. When network data are aggregated, anomalies can occur at any point in time. However, it is important to note that the only time any network surveillance method can signal the presence of an anomaly is at the end of an aggregation period.

We refer to $P_{r,r}^{0}$ and $P_{r,r}^{\prime }$ as the baseline and anomalous propensities of communication within community *r*, respectively. All nodes within the network follow baseline behavior at the start of the simulation, where $P_{r,r}=P_{r,r}^{0}$. The anomalies are introduced in only a single community which is referred to as Community #1. Following this point, the propensity of communication among the nodes within Community #1 increases and $P_{1,1}=P_{1,1}^{\prime }$. We refer to the time period at which the anomaly is introduced as the *shift time*. In our simulation study, the increase in *P*_1,1_ is sustained, meaning once *P*_1,1_ increases to $P_{1,1}^{\prime }$ it does not return to $P_{1,1}^{0}$. Our simulations included shifts of $P_{1,1}^{\prime }=(1+s)\times P_{1,1}^{0}$, where *s* represents the relative shift magnitude with *s* = 0.5 or 1.5. The degree heterogeneity parameters *θ*_*i*_ were randomly generated and then kept fixed in each of the individual simulations used to estimate each detection rate.

For aggregation levels higher than W = 1, an equal number of simulations were performed for all possible shift times associated with an aggregation period. The anomaly was introduced at shift times ranging from *t* = 840 to *t* = 859 to simulate situations where anomalies can occur at any point within an aggregation period. Taking W = 20 aggregation as an example, $C_{t=42}^{\left(20\right)}$ is calculated from 1 baseline and 19 anomalous *C*_*t*_ matrices if the anomaly occurs at *t* = 841. Similarly, 5 baseline and 15 anomalous *C*_*t*_ matrices make up $C_{t=42}^{\left(20\right)}$ when the anomaly occurs at *t* = 845. In the context of Priebe’s scan method, a false alarm is defined as a signal within 20 time periods after an assumed random shift time when in truth no such shift occurs. We refer the proportion of simulations which resulted in least one false alarm as the false alarm rate (FAR).

Timely detection of any anomalies is an important consideration as well, thus we limited our simulation to 20 networks post-shift for the detection of an anomaly. If Priebe’s scan method correctly detected the presence of an anomaly within these 20 time periods, the simulation run is designated as a success, otherwise it is designated as a failure. The proportion of the simulations which resulted in the successful detection of the anomaly is referred to as the detection rate. The detection rates directly reflect any change in the method’s performance due to different data aggregation levels. Other metrics such as true positive detection rates are appropriate as well, although these are more appropriate if the researcher’s main interest is to directly evaluate a method’s detection performance.

Given an anomaly has been successfully detected, we call the time it takes for the method to signal the *conditional signal delay*. The goal of prospective social network surveillance is to detect anomalies quickly. Practitioners are affected by both detection rate and the time it takes for the method to detect the anomaly. We note that the false alarm rate, the detection rate, and the conditional signaling delay are widely used performance metrics in the field of process monitoring.

Both network count data and binary data were included in our investigation. In general, binary data may be generated by converting counts into indicator variables equal to 1 in adjacency matrix cell (*i*, *j*) if the communications count at time *t* is greater than some threshold and zero otherwise. In our simulations, the conversion threshold was chosen to be 1, simply indicating whether communication had occurred or not. One could use a larger threshold to indicate whether or not a specified level of communication had occurred. The corresponding binary adjacency matrix is denoted by *B*_*t*_ or *B*_*t*_^(*W*)^. As with aggregating the count data, converting the observed counts into binary indicators directly removes the possibility that observed performance differences are due to simulation variation.

## False alarm rates

It is not meaningful to compare the anomaly detection ability of methods which do not have similar baseline performance, since it is always possible to improve the anomaly detection rate at the expense of more false alarms by decreasing the signal threshold. In order to be certain that the cause of any performance differences is only attributable to the aggregation levels, we need to be sure that the FAR is at least roughly the same under different levels of aggregation.

Using the signal threshold of 4, we generated *T* = 860 networks of order |*V*| = 20 with nodes evenly distributed into *R* = 2 communities. The *P* matrix has diagonal values ranging from $P_{r,r}^{0}=0.2$ to $P_{r,r}^{0}=5$, representing various levels of network sparsity. The off-diagonal values were $P_{r,r^{\prime }}^{0}=0.5\times P_{r,r}^{0}$. Times ranging from *t* = 840 to *t* = 859 were designated, and *P*_1,1_ was kept at the baseline value $P_{1,1}=P_{1,1}^{0}$ for the entire simulation. A total of 1000 simulations were performed at each aggregation level. We found that the estimated FARs were low for all aggregation levels and sparsity levels. There were only relatively small differences in the FARs for count versus binary data. These results imply that any variations in anomaly detection performance in our study are due to the aggregation levels. The detection rate results that we obtain from networks with different sparsity levels and network data types are thus comparable. It is important to note that practitioners can adjust the signaling threshold to achieve their preferred false alarm rate. Establishing the false alarm rate or some other measure of baseline performance is crucial in designing a monitoring method.

## Evaluation of detection rates

In this section, we describe the results of a simulation which evaluated the effect of temporal aggregation using both count and binary data, combined with a range of network sparsity levels and shift magnitudes. We considered networks of order |*V*| = 20, 50, and 100. Recall that for each combination of |*V*| and W, we carried out 1000 simulations, and each simulation involves 860 synthetic networks. This makes the simulation study computationally expensive, even after employing a distributed parallel architecture for scalability. It is mainly due to the computational burden that we did not use larger networks in our investigation.

### Effect of network sparsity level

The effect of aggregation can vary significantly between sparse networks and ones that are relatively non-sparse. To explore the relationship between aggregation and sparsity levels, we performed simulations using a wide range of $P_{r,r}^{0}$ and $P_{r,r^{\prime }}^{0}$ values. As described earlier, anomalous shifts to be detected were fixed at $P_{1,1}^{\prime }=(1+s)\times P_{1,1}^{0}$, where *s* = 0.5 and 1.5. The networks simulated were of order |*V*| = 20 with two communities, each containing 10 nodes. The signaling threshold was set to 4.

**Fig 1:**
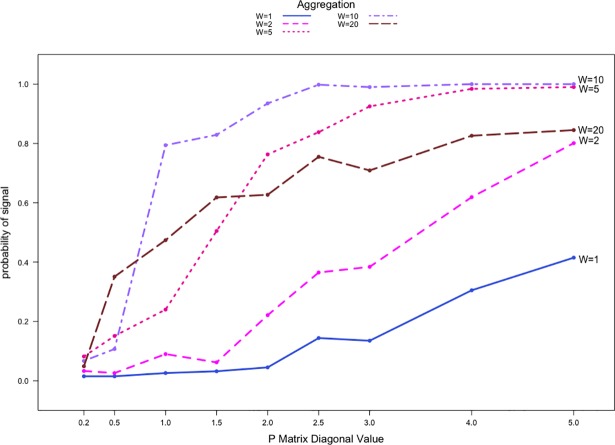
Detection rates against $P_{r,r}^{0}$ for count communication data with |*V*| = 20 and two communities. $P_{1,1}^{\prime }=(1+0.5)\times P_{1,1}^{0}$.

Fig 1 shows the detection rates at different temporal aggregation levels using count data with the shift $P_{1,1}^{\prime }=(1+0.5)\times P_{1,1}^{0}$. Each point represents the detection rate estimated from 2000 simulations. Fig 1 shows that Priebe’s scan method performs poorly for the case when $P_{r,r}^{0}=0.2$, where detection rates are less than 10% regardless of the aggregation level. For all aggregation levels, detection rates increase as $P_{r,r}^{0}$ increases. Overall, the performance of Priebe’s scan method is better for relatively non-sparse networks than sparse networks.

The most substantial improvement in performance is seen when the aggregation level increases from W = 2 to W = 5, with an increase of 50 percentage points in detection rates in some cases. Increasing the aggregation level from W = 5 to W = 10 also results in modest improvement in performance where the detection rate can increase by 20 percentage points. The detection rate is generally better for higher levels of temporal aggregation, with the exception of W = 20, where the performance is generally poorer than that at W = 10. In cases where the network is relatively non-sparse (i.e., when $P_{r,r}^{0}\geq 1.5$), the performance can be lower than that of the W = 5 aggregation level. When $P_{r,r}^{0}\geq 1.0$ and specifically for W = 20, the detection rates are 20 percentage points lower than that for W = 10. When $P_{r,r}^{0}\geq 1.5$, detection rates for the highest level of temporal aggregation are 10 to 20 percentage points lower than those for W = 5. For levels of aggregation higher than W = 20, the performance will continue to deteriorate under our assumptions.

An anomaly needs to be detected quickly after its occurrence so that practitioners can investigate the situation as soon as possible. The ability to detect anomalies in a timely manner is a quality that is as important as the detection rate performance for any network surveillance method. By design, our simulations extend past *t* = 840 by 20 adjacency matrices to reflect this practical scenario. Thus, as the aggregation level increases, the number of available post-anomaly adjacency matrices diminishes quickly. At the W = 20 aggregation level, Priebe’s scan method only has a single opportunity to detect the presence of an anomaly within the specified 20 time periods whereas the method has ten, four, and two chances for detection when W = 2, 5, or 10, respectively. Therefore, temporal aggregation at the highest W = 20 level has a distinct disadvantage. The resulting performance drop is not a direct result of aggregation at the W = 20 level, but a consequence of the limited data availability post-shift and the lack of signaling opportunity. We would see similar deterioration in performance for W = 5, or W = 10 if we required detection within 5 or 10 time periods. Additionally, the need to wait until the end of an aggregation period to signal an anomaly can delay detection. We explore conditional signal delays in Section 6.

We also studied the detection rates when binary data were used instead of count data. Although not shown here, the performance of Priebe’s scan method is significantly worse than the results shown in Fig 1. Priebe’s scan method was unable to detect any anomalies regardless of the sparsity or aggregation level. Detection rates were nearly 0% for all simulations with the poor performance attributed to the $P_{r,r}^{0}$ values used in the simulations. Our simulations using $P_{r,r}^{0}=0.2$ or larger result in relatively non-sparse *B*_*t*_ matrices for the networks generated; in other words, at any given time *t* we have *B*_*t*_(*i*,*j*) = 1 for nearly all *i* and *j* in the baseline networks. When an anomaly occurs, a high percentage of the elements in the *B*_*t*_ matrices representing communications among the anomalous nodes remain unity. Priebe’s scan method performs poorly in this case since there are no discernable differences between the baseline and anomalous *B*_*t*_ matrices.

**Fig 2:**
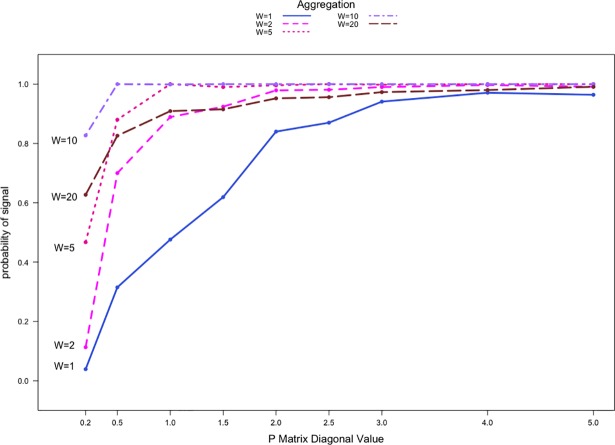
Detection rates against $P_{r,r}^{0}$ for count communication data with |*V*| = 20 and two communities. $P_{1,1}^{\prime }=(1+1.5)\times P_{1,1}^{0}$.

We also performed simulations with substantially more severe anomalies using *s* = 1.5. Fig 2 shows a similar pattern compared to Fig 1, where the detection rates increase as network sparsity level decreases. The performance is significantly better for all aggregation and sparsity levels in these simulations due to the larger shift. When comparing aggregation levels, the largest improvement is again seen when aggregation level increases from W = 2 to W = 5. In this case, detection rates increase by as much as 30 percentage points for relatively sparse networks. For data aggregated at W = 20, the detection rates, similar to Fig 1, are lower than those for W = 10 by 10 percentage points. They are, however, higher than the values for W = 5 across all sparsity levels. We see again that increasing the level of aggregation can improve performance up to a certain point, but the diminishing data availability post-anomaly from too much aggregation quickly leads to a decrease in performance. Although not shown here, our simulations also revealed that for the shift $P_{1,1}^{\prime }=(1+1.5)\times P_{1,1}^{0}$, the detection rates for |*V*| = 50 networks are comparable to those in Fig 2.

We obtained detection rates with the use of binary data as well for the shift of $P_{1,1}^{\prime }=(1+1.5)\times P_{1,1}^{0}$. The overall detection rates improved slightly due to the larger shift, ranging between 2% to 10% across all aggregation and network sparsity levels. However, for both shift magnitudes the results showed that there is a significant loss of information in situations where binary data were used in place of count data. Performance may have been improved if a higher threshold had been used to define the binary data.

**Fig 3:**
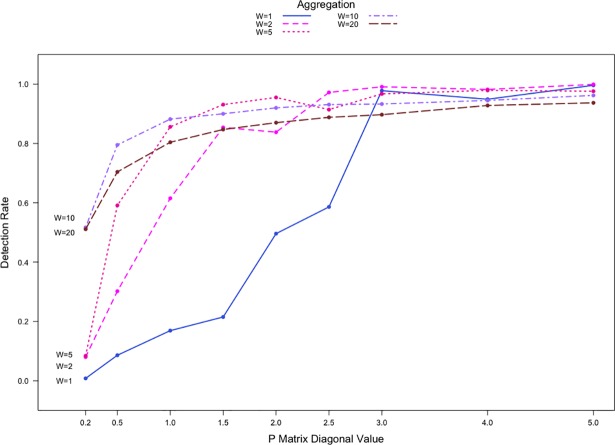
Detection rates against $P_{r,r}^{0}$ for count communication data with |*V*| = 50 and two communities. $P_{1,1}^{\prime }=(1+0.5)\times P_{1,1}^{0}$.

In addition to simulating networks of order |*V*| = 20, we simulated larger networks of order |*V*| = 50 and |*V*| = 100. Fig 3 illustrates the detection rates at different temporal aggregation levels for count data with the shift $P_{1,1}^{\prime }=(1+0.5)\times P_{1,1}^{0}$ where the networks are of order |*V*| = 50. The networks contain two equally sized communities each made up of 25 nodes. Each point represents the average detection rate from 2000 simulations. Fig 3 shows a similar detection rate pattern compared to that of the |*V*| = 20 networks. Priebe’s scan method again performs poorly for $P_{r,r}^{0}=0.2$ with the detection rates for W = 1, 2, and 5 less than 10% and at 50% compared to W = 10, 20. The performance improves across all aggregation levels as network sparsity level decreases. When comparing the aggregation levels, the most significant improvement in performance, with detection rates as much as 40% higher, is now observed when the aggregation increases from W = 1 to W = 2. The detection rates for W = 20 are consistently lower than that for W = 10, for all sparsity levels. When $P_{r,r}^{0}\geq 1.0$, detection rates for W = 10 are lower than those for W = 5.

Fig 3 shows somewhat different patterns for the detection rates compared to Fig 2. Note that a node within a larger network (e.g., with |*V*| = 50) can be in communication with considerably more nodes compared to a node within a smaller network (e.g., |*V*| = 20) at any given time *t*. Thus, when |*V*| = 50, there is a significant increase in baseline numbers of communications among nodes during *Phase I* since an individual node has 49 potential targets with which to establish a connection. For shifts of identical magnitude, it is more difficult for Priebe’s scan method to detect the presence of an anomaly relative to when |*V*| = 20. For the |*V*| = 50 networks in our simulations, W = 5 represents the most reasonable balance between amplifying the severity of the anomaly and giving the method ample opportunity for detection.

**Fig 4:**
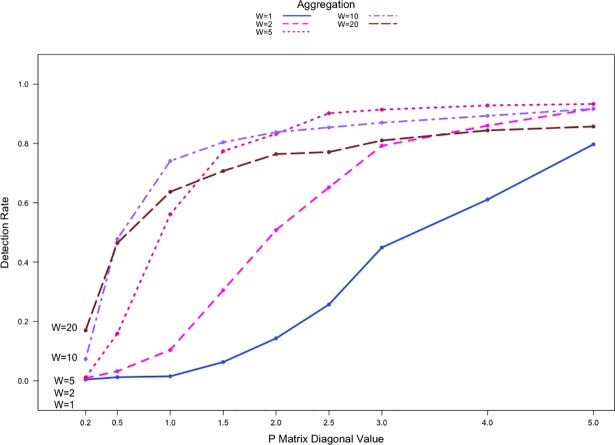
Detection rates against $P_{r,r}^{0}$ for count communication data with |*V*| = 100 with four communities. $P_{1,1}^{\prime }=(1+0.5)\times P_{1,1}^{0}$.

The detection rates from the use of count data in networks with order of |*V*| = 100 are shown in Fig 4 with the shift set at $P_{1,1}^{\prime }=(1+0.5)\times P_{1,1}^{0}$. In this case the networks contain four equally sized communities with 25 nodes each. We observe similar patterns in detection rates when compared to Fig 2 and Fig 3. However, regardless of the aggregation level or network sparsity, the detection rates in Fig 4 never approach 100%. Clearly, the relative order of a community within a network affects the performance of the method. This is consistent with the findings in Zhao et al. [25], where the performance of Priebe’s scan method can be poorer for small anomalous sub-networks.

To summarize, our results show that there is a notable drop in performance when W = 20. The decrease in performance can be attributed to the lack of availability of post-shift adjacency matrices and the long aggregation period. While aggregating network data can increase the detection rate, the optimal aggregation level should be chosen by considering factors such as data availability and the speed of detection. The performance of Priebe’s scan method is much better when count data are used. Use of count data offers practitioners a clear advantage over the use of binary data. Even still, we further examine the influence of data aggregation on the performance of Priebe’s method in the binary situation in Section 5.3.

### Effect of anomaly magnitudes

The severity of the anomaly is modeled by the shift magnitude, a factor that can significantly affect the performance of Priebe’s scan method. We study how shift magnitudes change the effects of aggregation in this section. Using the shift $P_{1,1}^{\prime }=(1+s)\times P_{1,1}^{0}$, where *s* = 0.05,0.25,0.5,1, and 1.5, networks of order |*V*| = 20 were generated, again with the nodes evenly divided into two communities. The baseline diagonal value of *P* is fixed at $P_{r,r}^{0}=1$.

**Fig 5:**
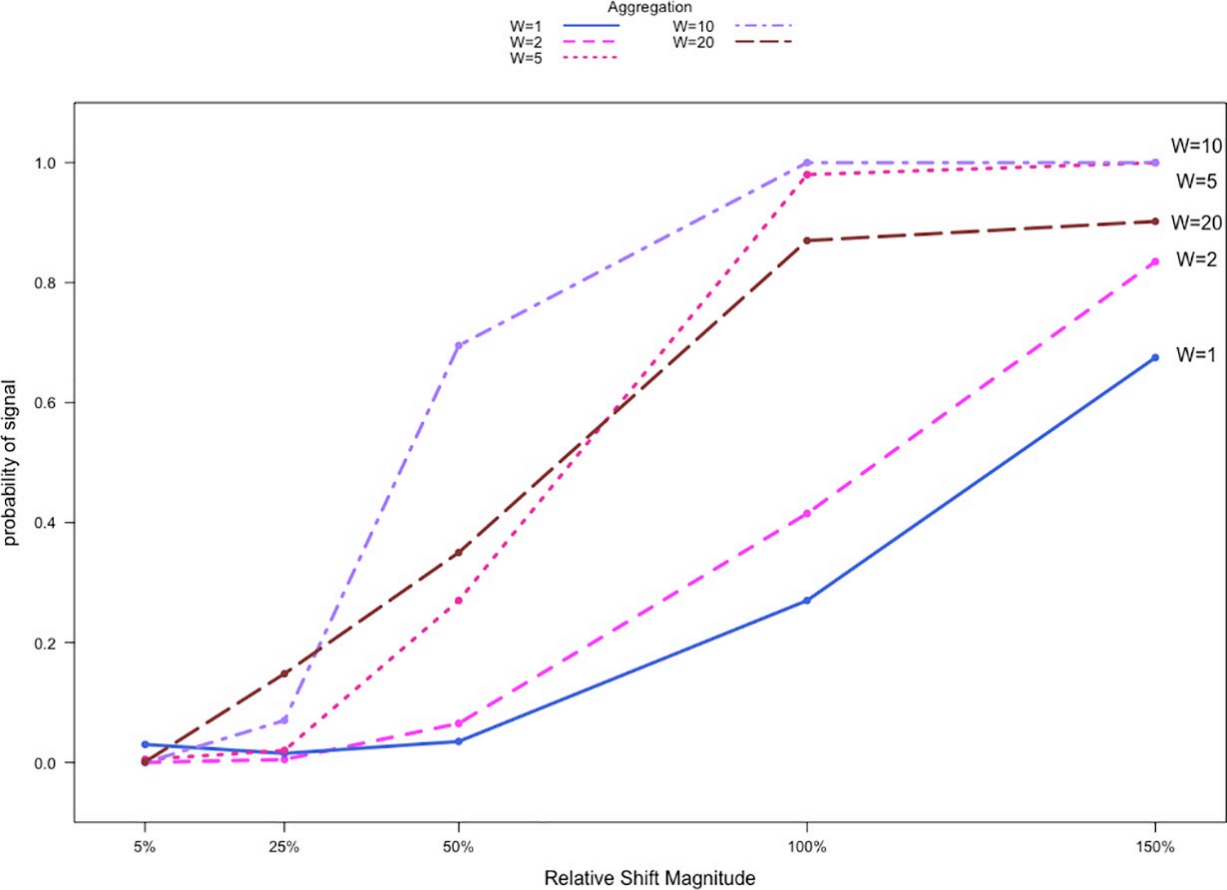
Detection rates against shift magnitudes for network count communication data. W = 20, P_*r*,*r*_ = 1.

Fig 5 shows detection rates for network count data aggregated at various levels for different values of *s*, where *s* represents the relative shift magnitude in the value of $P_{1,1}^{0}$ in our simulations. When *s*≤0.25, the detection rates are low for all aggregation levels. When *s*>0.25, aggregation increases the detection rate. Generally, the detection rates are higher for larger shifts. Studying the results from using data aggregated at different levels reveals that increasing W from 2 to 5 leads to the biggest improvement in detection rates, echoing the results in Fig 3 and Fig 4. Similar to results discussed in Section 5.1, when 0.50≤*s*≤0.75, detection rates for W = 20 are 20 to 40 percentage points lower than those for W = 10. When *s*≥1, the detection rates for W = 20 are 10 to 20 percentage points lower than those for W = 5. Aggregating at the W = 20 level again shows lower performance resulting from the limited post-shift data availability and the single opportunity for detection. While results from the use of binary data are not shown here, detection rates were nearly 0% regardless of the aggregation level after converting the network data to binary form, reinforcing the point that using binary data can lead to a significant loss of information when networks are not sparse.

### Simulations for binary network data

The results discussed in Sections 5.1 and 5.2 indicate that converting network count data into binary data results in considerably lower detection rates. Use of binary data is not as efficient compared to the use of count data in network surveillance applications. The simulation settings used, however, were not helpful for studying the effect of aggregation using binary network data so we consider other settings in this section. Because the conversion from network count data to binary data is not linear, the same anomaly severity specified in the simulation for count data does not yield the same effect on binary data. In this section, we compare the expected change in *B*_*t*_ pre- and post-shift in order to find applicable simulation settings for the study of the use of binary data. Specifically, network densities are used for comparison, where density is defined as the proportion of the potential connections in a network that are actual connections.

**Fig 6:**
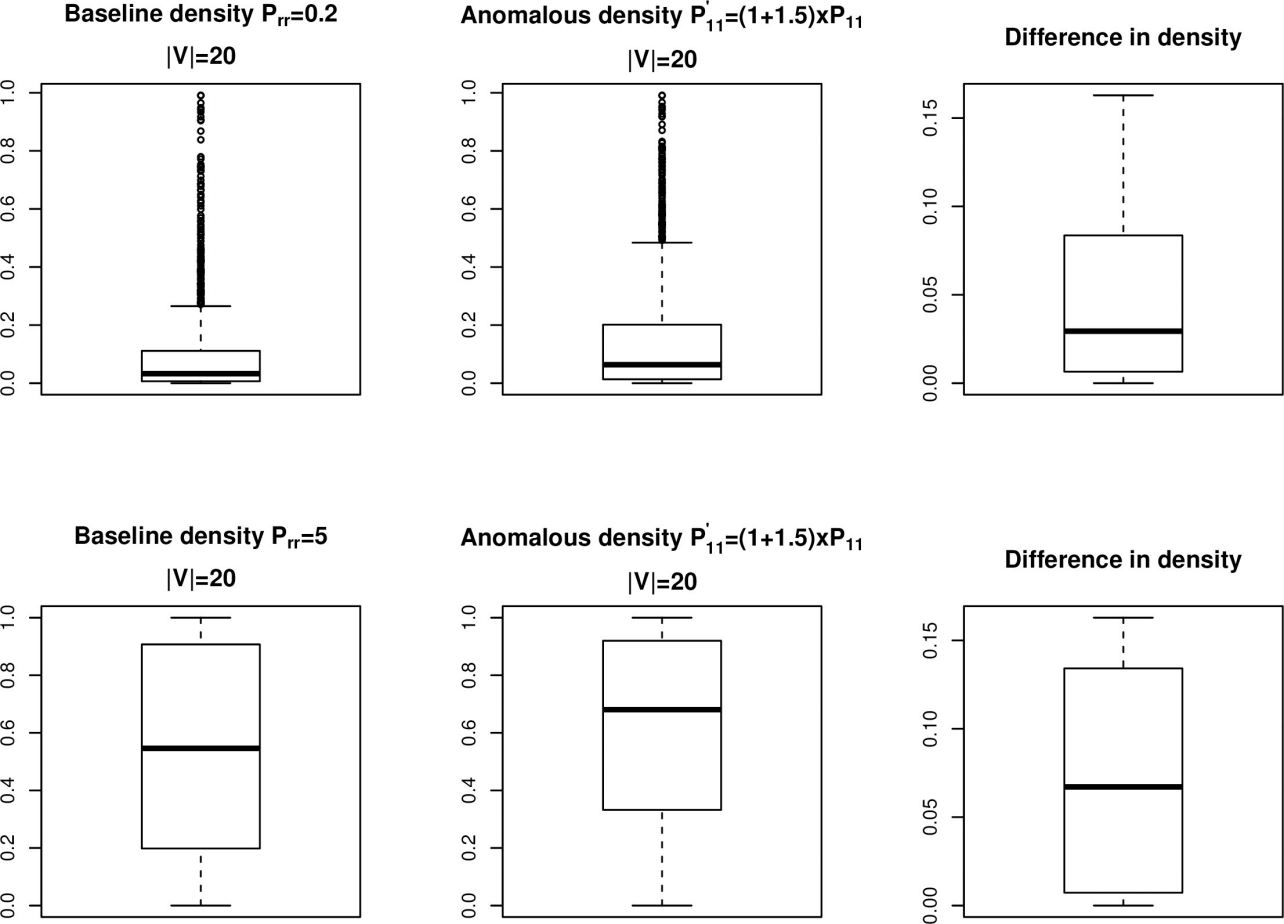
Network densities pre- and post-shift. Binary networks with |*V*| = 20 and two communities. Shift magnitude is +150%.

**Fig 7:**
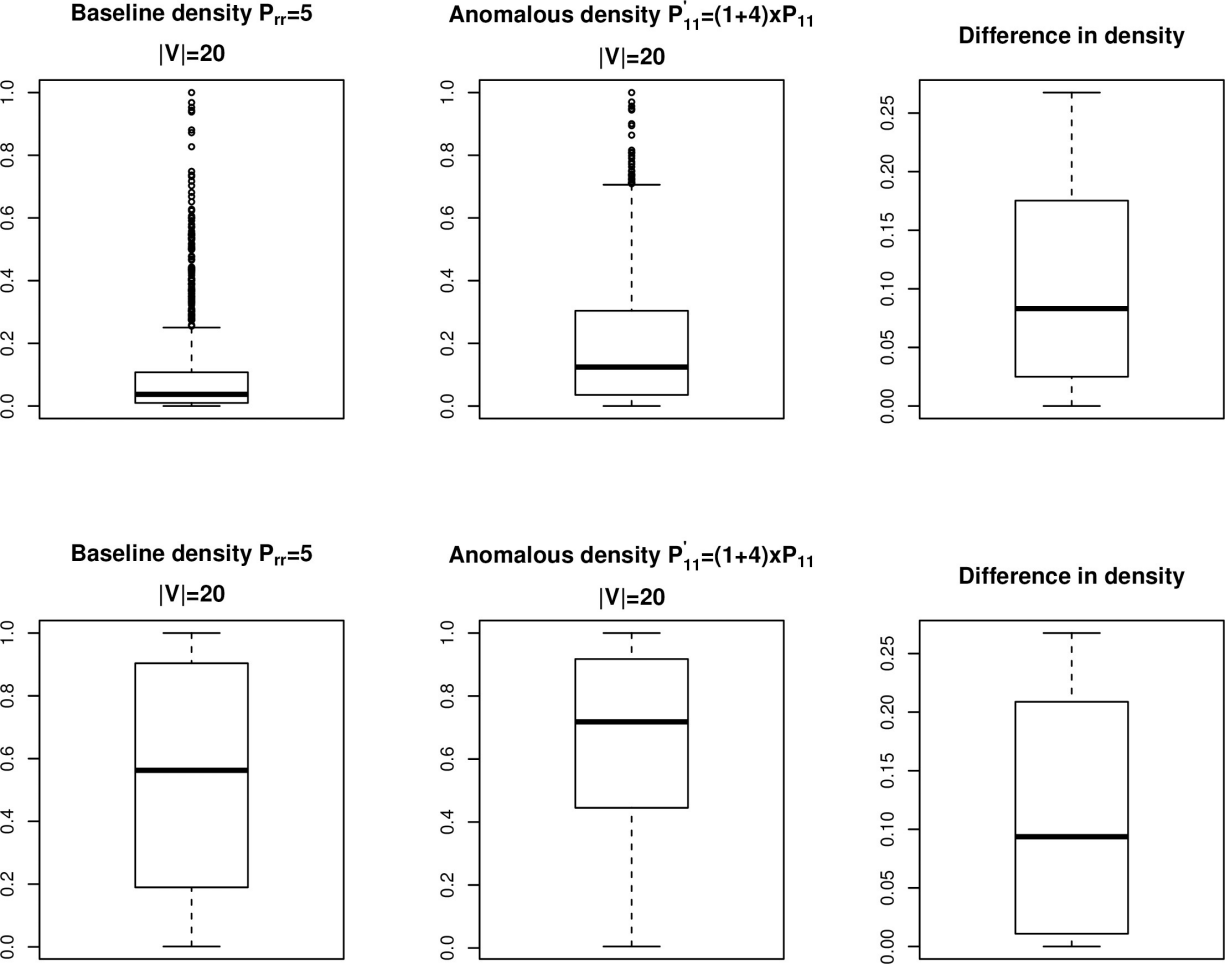
Network densities pre- and post-shift. Binary networks with |*V*| = 20 and two communities. Shift magnitude is +400%.

We generated 2000 networks of order |*V*| = 20 each with two equally sized communities, where $P_{1,1}^{\prime }=(1+1.5)\times P_{1,1}^{0}$, and $P_{1,1}^{\prime }=(1+4)\times P_{1,1}^{0}$. Fig 6 shows the distribution of network densities for *B*_*t*_ pre- and post-shift and at two different sparsity levels. In both cases, the changes in network densities are not affected by the sparsity level. The pre- and post-shift values only differ by up to about 15%, not the +150% as specified in the simulation for the *P*_1,1_ component. Fig 7 shows the distribution of network densities for *B*_*t*_ pre- and post-shift and at two different sparsity levels in the case of $P_{1,1}^{\prime }=(1+4)\times P_{1,1}^{0}.$ The difference in the densities are only up to slightly more than 25%. In order to detect anomalies reliably with binary data, the difference between the pre- and post-shift densities needs to be significantly higher. As such, $P_{r,r}^{0}$ has to be much smaller while *s* needs to be much larger. In other words, the relative severity of the anomalies needs to be much stronger with the use of network binary data in order for Priebe’s scan method to detect the anomalous behavior.

**Fig 8:**
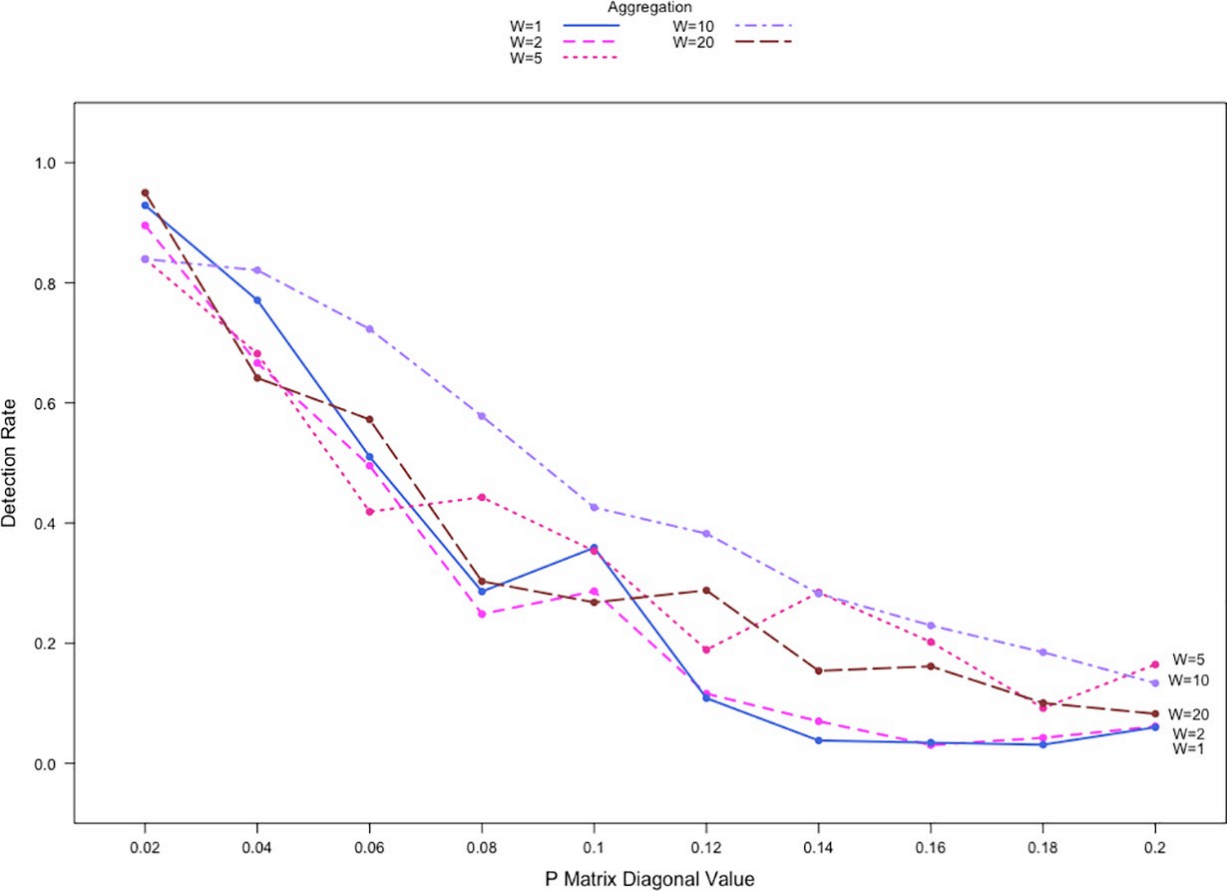
Detection rates against *P*_*r*,*r*_ for |*V*| = 20 binary networks with two communities. $P_{1,1}^{\prime }=(1+s)\times P_{1,1}^{0}$, *s* = (5,50).

Consequently, we simulated networks with $P_{r,r}^{0}$ ranging from 0.02 to 0.2. Anomalies were simulated with much larger shift magnitudes for the propensity of communication within community #1, with $P_{1,1}^{\prime }=(1+s)\times P_{1,1}^{0}$. We set a fixed value for $P_{1,1}^{\prime }=1$ and this results in *s* ranging from 5 to 50 with the various values of $P_{1,1}^{0}$. A total of 2000 simulations were performed for each combination of *s* value and aggregation level. By adopting a wide range of *s* values in our simulations, we are able to vary the difference between the pre- and post-shift network density significantly. Fig 8 shows the detection rates by aggregation levels at different $P_{r,r}^{0}$ values. When $P_{r,r}^{0}=0.02$, Priebe’s scan method is able to detect the presence of an anomaly with a reliable consistency. Detection rates range from 80% to 100% depending on the aggregation level. When $0.04\leq P_{r,r}^{0}\leq 0.12$, detection rates decrease significantly, by as much as 50 percentage points. The same effect is observed regardless of the aggregation level. This is because as $P_{r,r}^{0}$ increases, there are more baseline node-to-node interactions, i.e. *B*_*t*_(*i*,*j*) = 1 occurs more often pre-shift. The change in the network densities post-shift in turn is small, making anomaly detection more difficult. When comparing different aggregation levels, we see that there is little difference in detection rates among different aggregation levels. Aggregation in this situation often results in $C_{t}^{\left(W\right)}(i,j)\geq 1$, which is then converted to $B_{t}^{\left(W\right)}(i,j)=1$ using our conversion threshold. The resulting $B_{t}^{\left(W\right)}$ matrices are very similar regardless of the aggregation level. This similarity reduces the effect of aggregation. We performed simulations using network count data as well. While not shown, the performance with the use of count data for all *s* values is much higher with detection rates that are generally at 95% and frequently above 99%.

## Distribution of conditional signal delay

**Fig 9:**
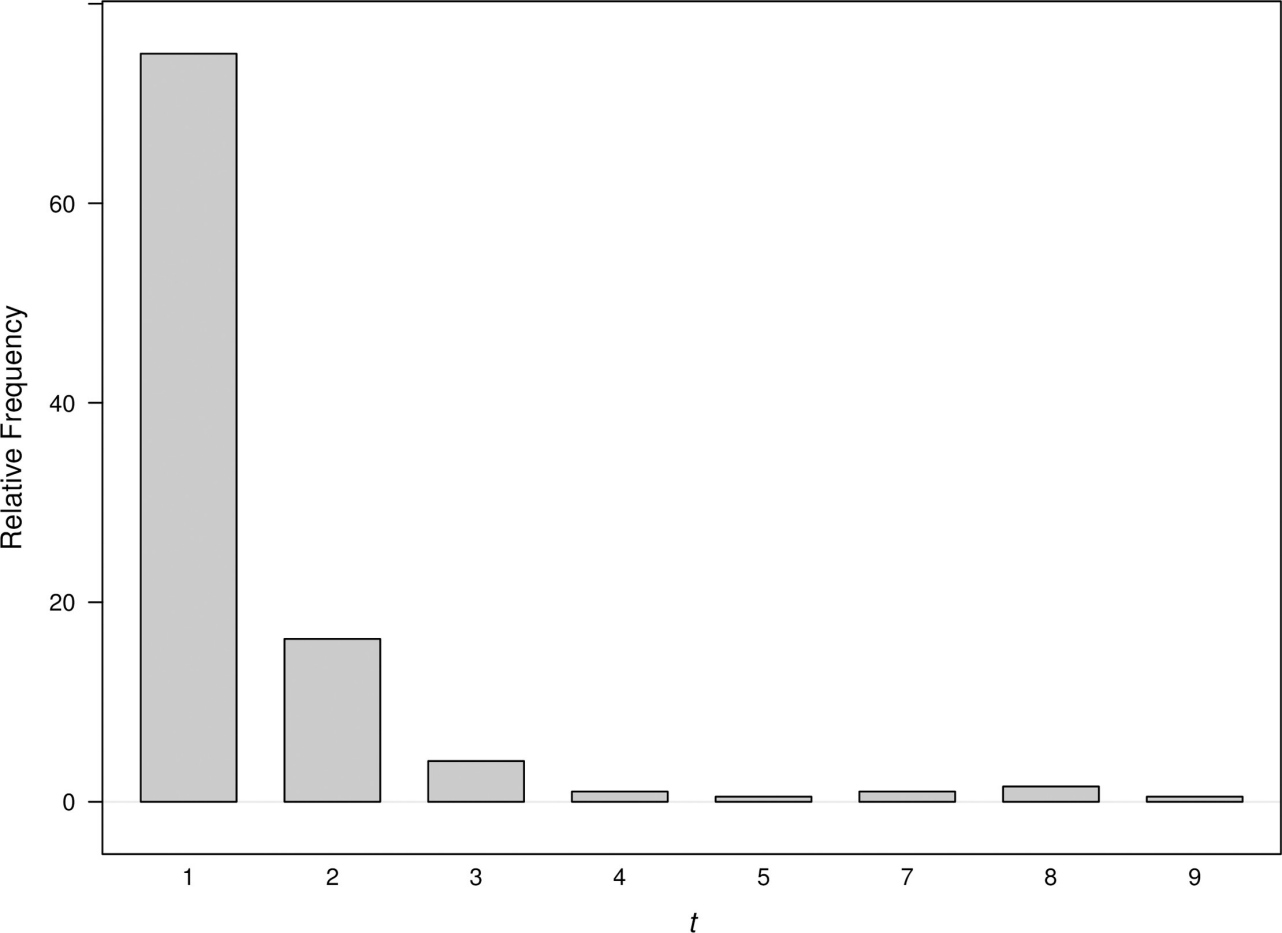
Distribution of conditional signal delay for network count data aggregated at W = 1 level. $P_{1,1}^{\prime }=(1+1.5)\times P_{1,1}^{0}$.

In this section we investigate the inherent delay due to temporal aggregation by studying the conditional delays when monitoring aggregated count data. Performing the same simulations as described in Section 5, we generated networks of order |*V*| = 20 with nodes evenly distributed into two communities. While simulating anomalies using $P_{1,1}^{\prime }=(1+1.5)\times P_{1,1}^{0},$ we recorded the conditional signal delay from the proportion of 1000 simulations where the anomalies were detected successfully. Fig 9 shows the distribution of conditional signal delay for the W = 1 aggregation level. We see that there is most often no delay in signaling. Detection is almost immediate because there is no need to wait until the end of a long aggregation period. A large majority of signals occur within two time periods following the shift given that there is a signal.

**Fig 10:**
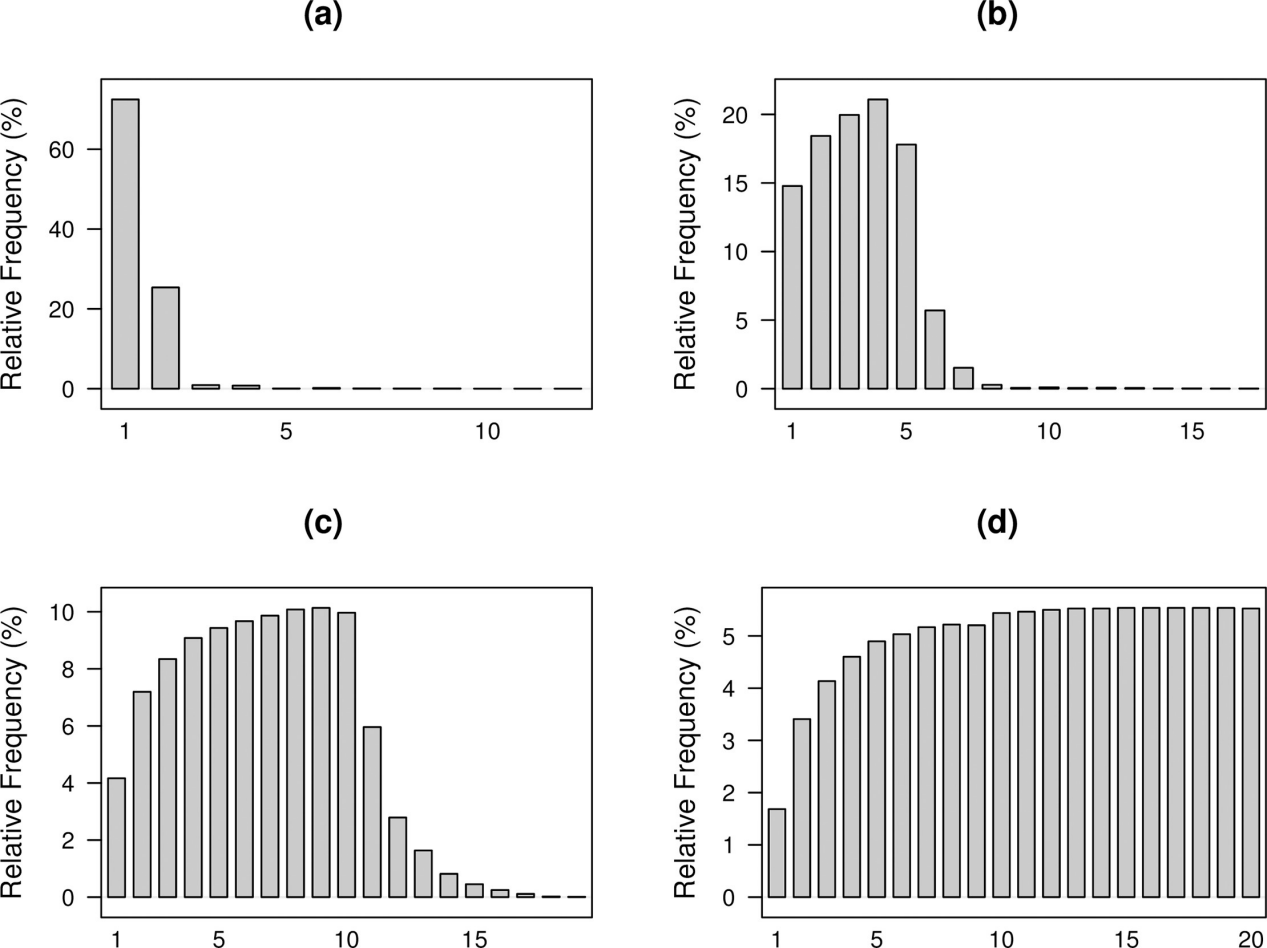
Distribution of conditional signal delay for network count data aggregated at (a) *W* = 2 (b) *W* = 5 (c) W = 10 and (d) *W* = 20 level. $P_{1,1}^{\prime }=(1+1.5)\times P_{1,1}^{0}$.

Larger conditional signal delays can be unavoidable at higher aggregation levels. Fig 10 shows the conditional signal delay for W = 2, 5, 10, and 20. For W = 2, anomaly detection took slightly longer in a few cases. For even higher aggregation levels, we see that signaling progressively took longer as aggregation levels increased. For W = 20, over half of the signals occur more than ten time periods following the shift. Aggregation levels that are higher than 20 will cause even greater signaling delays when signals occur. Fig 9 and Fig 10 reflect the fact that if the scan method signals an anomaly, then it tends to do so at the first opportunity after the anomaly occurs.

**Fig 11:**
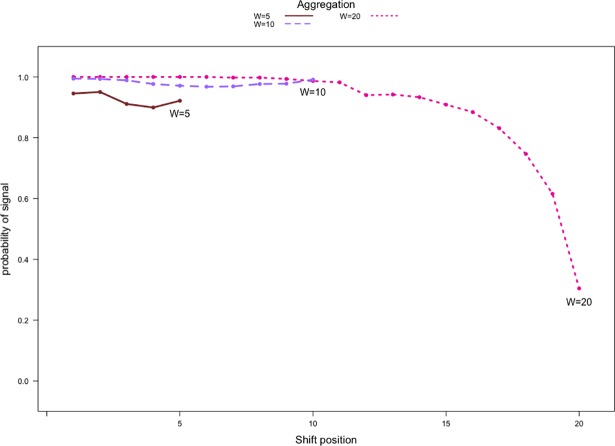
Detection rates against shift time within the aggregation period for communication count data from |*V*| = 20 networks with 2 communities. $P_{1,1}^{\prime }=(1+1.5)\times P_{1,1}^{0}$.

It is important to stress that for immediate detection with Priebe’s scan method, a large amount of the aggregated data within an aggregation period must be anomalous. Depending on when an anomaly occurs within an aggregation period, performance could be negatively affected, especially for W = 20. With W = 20, there are 20 possible shift times within an aggregation period. If the shift occurs during the latter part of the aggregation period, only a small portion of the aggregated data will be anomalous. Fig 11 shows how shift times affect the performance with respect to each aggregation level. Both W = 5 and W = 10 result in lower detection rates if anomalies occur during the second half of the aggregation period compared to the beginning of the aggregation period. Moreover, we see a slight increase in detection rates immediately after the performance drop. The recovery in performance is attributed to the fact that the aggregation period immediately after consists entirely of anomalous data, giving the method another chance to signal. At W = 20, we see that if anomalies occur at or after the 12^th^ adjacency matrix in a window of 20 within the aggregation period, the performance starts to decrease, and continues to do so until the detection rate drops to 25%. In this case, Priebe’s scan method only has one chance to signal within 20 time periods due to the high level of aggregation and cannot detect the anomalies reliably when less than 40% of the data within the aggregation period are anomalous. This phenomenon will be more pronounced with higher aggregation levels.

## Conclusions and directions for research

We have demonstrated how aggregating social network data at different levels affects the performance of Priebe’s scan method. Our results show that aggregating network data at higher levels can be beneficial to the detection rate performance of Priebe’s scan method up to a certain point before becoming detrimental. Additionally, the simulation studies show that there is a significant decrease in performance due to information loss in situations where binary data are used in place of count data.

By studying the conditional signal delay and the relationship between detection performance and shift time, we are able to attribute the poor performance for high levels of aggregation to two causes. First, when there is a limited time window allowed for detection post anomaly, the number of available adjacency matrices diminishes quickly as the aggregation level increases. At a certain level, it is likely that the network surveillance method used only has a single opportunity to detect the presence of an anomaly within a specified time window. Second, depending on the aggregation level, there are a number of possible shift times within an aggregation period. If an anomaly occurs during the latter part of an aggregation period, only a small portion of the aggregated data are anomalous. Successful immediate detection is made very difficult in these situations. Practitioners must wait until the end of the next aggregation period to determine if an anomaly is present.

We recommend the use of count data to summarize the level of interaction between nodes in a network, if available, rather than binary data. Moreover, we recommend the use of aggregation at an appropriate level for network surveillance applications. The specific aggregation level used depends to a large extent on the signal delay that can be tolerated in the particular monitoring situation.

We consider only one combination of network model, surveillance method, and anomaly in our study, but other combinations should be studied. Network anomalies might be transient instead of sustained, as we assumed, with an expected large effect on the appropriate level of aggregation. In addition, further investigation is needed into the performance of network surveillance techniques when count data are converted to binary data using a higher conversion threshold.

Our investigation is based on synthetic networks. In application data, there can be additional practical challenges related to temporal aggregation, and here we briefly discuss a few of them. Depending upon the origin of the data, a practitioner might not have the option of using aggregation levels smaller than a certain width. Also, in this paper we assumed that the most basic level of aggregation has removed any seasonal variation, which might not be true in practice. We also assumed that the community structure is known, which may not hold true in practice. Finally, we generated temporal sequences of social networks as independent realizations from the DCSBM model. In practice, there can be dependence in the temporal sequence of social networks.

Communications in social networks often take place on a continuous time scale rather than a discrete time step. But currently available methods for social network surveillance are largely designed for discrete time steps, which implies that the network data must be aggregated over some time period before surveillance can be conducted. This step of aggregation can lead to a costly loss of information. In future work, we aim to address this issue by developing methods for social network surveillance that can be implemented on a continuous time scale.

In future work, we also plan to investigate networks with larger orders than the ones studied in this paper, and consider anomalous events where the nodes affected are not necessarily in the same community.
